# Presence of ice-nucleating *Pseudomonas* on wheat leaves promotes Septoria tritici blotch disease (*Zymoseptoria tritici*) via a mutually beneficial interaction

**DOI:** 10.1038/s41598-020-74615-7

**Published:** 2020-10-20

**Authors:** Helen N. Fones

**Affiliations:** Biosciences, University of Exeter, Stocker Road, Exeter, EX4 4QD UK

**Keywords:** Plant sciences, Plant ecology, Microbial ecology, Microbiome, Symbiosis, Bacteria, Fungi

## Abstract

*Zymoseptoria tritici* causes Septoria tritici blotch (STB) of wheat, an economically important disease causing yield losses of up to 10% despite the use of fungicides and resistant cultivars. *Z. tritici* infection is symptomless for around 10 days, during which time the fungus grows randomly across the leaf surface prior to entry through stomata. Wounded leaves show faster, more extensive STB, suggesting that wounds facilitate fungal entry. Wheat leaves also host epiphytic bacteria; these include ice-nucleating (INA+) bacteria, which induce frost damage at warmer temperatures than it otherwise occurs. Here, STB is shown to be more rapid and severe when wheat is exposed to both INA+ bacteria and sub-zero temperatures. This suggests that ice-nucleation-induced wounding of the wheat leaf provides additional openings for fungal entry. INA+ bacterial populations are shown to benefit from the presence of *Z. tritici*, indicating that this microbial interaction is mutualistic. Finally, control of INA+ bacteria is shown to reduce STB.

## Introduction

*Zymoseptoria tritici* is the causal agent of Septoria tritici blotch (STB), an economically important disease of temperate wheat^1^. Even the most STB resistant wheat varieties require 2–3 applications of fungicide, costing the UK economy around £150 M annually^1^, to reduce STB-related yield losses to around 10%^1^. UK winter wheat is sown in the autumn, with foundation growth occurring during winter and rapid increase in biomass beginning around mid-April with the onset of stem elongation [Growth stage (GS) 31]^2^. Fungicides are first applied at GS31–32^3^. This is much later than the first STB symptoms, however, which are observed in autumn^3^. Although the most severe losses occur if STB affects the flag leaf during grain filling, the polycyclic nature of *Z. tritici* infection means that early infection can be an important factor in later disease^2–4^.

*Zymoseptoria tritici* infection is symptomless for around 10 days, during which time the fungus is assumed to act as a biotroph^5–7^, although without feeding structures. However, recent work indicated that some strains may in fact be largely epiphytic throughout the symptomless phase^8,9^. Following ingress via stomata, the fungus colonises the leaf, before inducing a strong anti-biotrophic response from the plant which it hijacks for necrotrophic growth^10^. The epiphytic/biotrophic phase is relatively poorly understood, but surface growth is known to be random and humidity-dependent^8^. Transcriptomic studies indicate the expression of cutinase, lipase and peptidase genes during this period, but not genes encoding plant cell wall degrading enzymes^7,10–13^.

Fones et al*.*^8^ demonstrated that wounded leaves are more susceptible to *Z. tritici*, and show fungal sporulation more rapidly. They postulated that wounds provide additional entry points for randomly growing hyphae, accelerating fungal ingress and consequently reducing the symptomless period. Wounding by herbivore, mechanical, or frost damage is a perpetual risk for crops. Frost damage is particularly relevant for winter wheat because of its growth throughout winter and early spring^2^. Such damage may be facilitated by the presence of ice-nucleating (INA+) bacteria^14–16^. INA+ bacteria, often epiphytes or opportunistic plant pathogens^17^, induce formation of ice crystals at slightly sub-zero temperatures. It has been hypothesised that ice-nucleation is an adaptation facilitating pathogenic bacterial ingress or nutrient acquisition via wounds^18^.

In this work, the hypothesis that frost damage induced by INA+ bacteria facilitates leaf entry by *Z. tritici* is tested, and the possibility of STB control via control of INA+ bacteria is explored. In addition, populations of INA+ and INA− bacteria are compared on frost-treated leaves in the presence or absence of *Z. tritici*, exploring the possibility of a mutualistic relationship to inform future studies of co-evolution between co-occurring pathogens.

## Results

Frost damage, detectable as increased ion leakage from leaves, was shown to occur when wheat leaves were briefly exposed to − 2 or − 5 °C in a frost chamber. Freezing treatments led to visible, light frost on leaves. Ion leakage from leaves increased significantly after freezing, compared to unfrozen controls. The degree of damage appeared to increase with freezing severity, but this effect was not significant (Supplementary Figure S1). To test the hypothesis that frost damage would promote STB, as seen for mechanical damage^8^, wheat leaves were subjected to 2 or 5 min exposure to − 2, − 4, − 5 or − 6 °C prior to inoculation with *Z. tritici*. At 14 days post inoculation (dpi), more advanced STB symptoms were seen in leaves subjected to the more severe treatments; leaves exposed to − 6 °C already showed pycnidia (Supplementary Figure S2).

**Figure 1:**
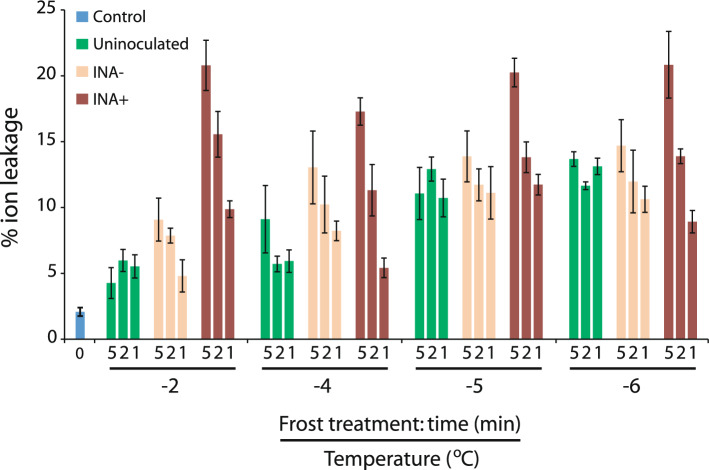
**INA+ bacteria induce increased ion leakage at slightly sub-zero temperatures**. Wheat leaves were inoculated with INA+ or INA− *Pseudomonas* or mock inoculated (10 mM MgCl_2_ only) prior to 1, 2 or 5 min exposure to − 2, − 4, − 5, or − 6 °C, and ion leakage then measured. Untreated controls were also measured. Values are means of three independent measurements, each consisting of 2–3 treated leaves from each of three pots of plants, pooled; error bars show SE. In per-temperature comparisons, treatment time was always significant (two-way ANOVAs: P < 0.0005 at − 6, − 4 and − 2 °C; P = 0.00054 at − 5 °C) and inoculum type was significant (P < 0.0005) at all temperatures except − 6 °C. The two factors did not interact significantly except at − 2 °C (P = 0.0024). In per-inoculum comparisons, temperature was always significant (two-way ANOVAs: P < 0.005 for both bacteria and P < 0.0001 for control) and time was significant for both bacteria (P = 0.014 for INA− and P < 0.0001 for INA+); these factors did not interact significantly for any inoculum.

To test the effect of INA+ bacteria upon frost damage, wheat leaves were inoculated with INA+ or INA− *Pseudomonas,* or mock inoculated, and maintained under normal growth conditions for 24 h before 1, 2 or 5 min exposure to sub-zero temperatures. Ion leakage was then measured. As previously, freezing treatment increased ion leakage (Fig. 1). In mock-inoculated leaves, lower temperatures increased ion leakage, but longer exposure time did not, while in INA− and INA+ inoculated leaves, both factors were significant. Consideration of the results on a temperature-by-temperature basis shows that at the coldest temperatures (− 6 or − 5 °C), inoculum and time were of relatively small importance, since even mock-inoculated leaves showed a 5–6 × increase in ion leakage over control (Fig. 1). At − 2 °C, however, ion leakage was significantly increased by INA+ bacteria, longer treatments, and the interaction between these (Fig. 1); ion leakage increased by up to 5 × for INA+ inoculated leaves, compared to uninoculated leaves, at this temperature. Further, for leaves treated at − 2 °C for 2 min, Bonferroni-corrected *t *tests show a significant (P = 0.014) difference between INA+ and INA− inoculation, but not (P = 0.13) between INA− and control. This frost treatment was therefore selected for further experimentation.

**Figure 2:**
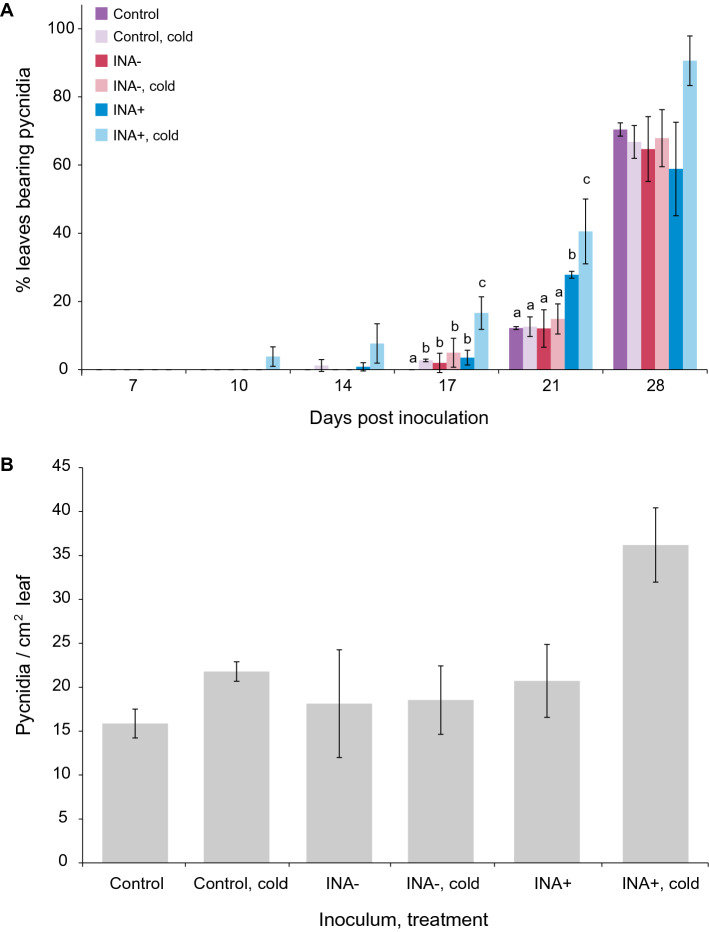
**Effect of ice-nucleating bacteria and frost treatment on percentage of leaves inoculated with** ***Z. tritici*** **showing pycnidia at various time points after infection** (**A**) **and final pycnidial density** (**B**). Wheat leaves were inoculated with MgCl_2_ only (control), or with either non ice-nucleating bacteria (INA−) or ice-nucleating bacteria (INA+) in MgCl_2_. Three to five leaves in each of 24 pots (total 72–120 leaves) were used in each treatment. After 24 h, 12 randomly selected pots from each treatment (36–60 leaves) were exposed to − 2 °C for 2 min. All plants were then inoculated with *Z. tritici* blastospores at 10^5^ cfu/mL and infected plants maintained as normal. Leaves were inspected and numbers of leaves showing pycnidia recorded every 3–4 days from 7 to 21 dpi (**A**). At 28 dpi, leaves were harvested and pycnidia enumerated (**B**). Values are means of two independent experiments and error bars show standard error. Letters above bars indicate significant (α = 0.05) differences between treatments (ANOVAs with Tukey’s simultaneous comparisons) and only apply within time points in (**A**). No letters are shown where the ANOVA result was non-significant (**A**: 7, 10, 14 and 28 dpi, and **B**).

To determine whether INA+ bacteria-induced frost damage to wheat leaves increases the speed of STB symptom development*,* wheat leaves were inoculated with INA+ or INA− *Pseudomonas,* or mock-inoculated, and maintained under normal growth conditions for 24 h, then frost-treated and inoculated with *Z. tritici.* The percentage of inoculated leaves showing pycnidia was recorded at various days post inoculation (dpi) (Fig. 2A) and pycnidia enumerated at 28 dpi (Fig. 2B). Leaves with INA+ bacteria and frost treatment were the first to show pycnidia, beginning at 10 dpi. Such leaves showed pycnidia on a significantly higher percentage of infected leaves than any other treatment at 17 and 21 dpi, although pycnidia were present in all treatments by 21 dpi. At 21 dpi, a small increase was also seen for leaves inoculated with INA+ bacteria but not cold-treated (Fig. 2A). By 28 dpi, the number of leaves bearing pycnidia was high in all cases, with no significant differences among treatments, although the INA+, frost treated leaves showed the highest percentage pycnidiation. Pycnidia counts reflected this, with a discernible but non-significant increase in numbers for INA+, frost treated leaves, and no differences among the other treatments (Fig. 2B).

**Figure 3:**
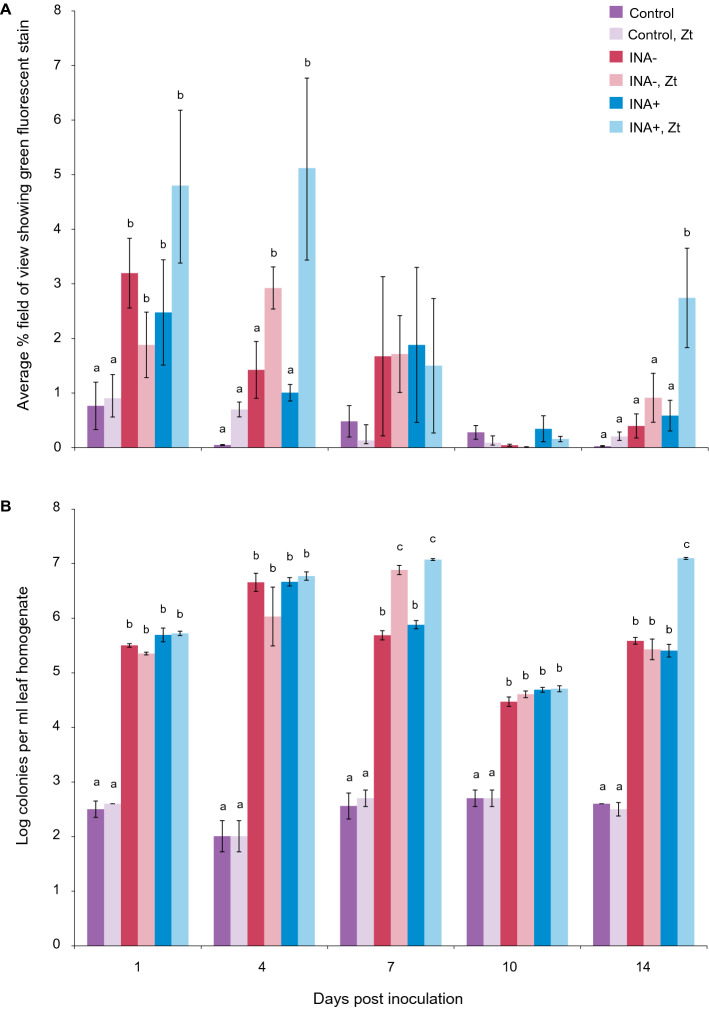
**Effect of** ***Z. tritici*** **co-inoculation on populations of INA+ and INA− bacteria on wheat leaves**. Wheat leaves were inoculated with MgCl_2_ only (control), or with either non ice-nucleating bacteria (INA−) or ice-nucleating bacteria (INA+) in MgCl_2_. Three to five leaves in each of 24 pots (total 72–120 leaves) were used in each treatment. After 24 h, all plants were exposed to − 2 °C for 2 min. Twelve randomly selected pots from each treatment (36–60 leaves) were then inoculated with either *Z. tritici* blastospores at 10^5^ cfu/mL or water (mock) and infected plants maintained as normal. At 3–4 day intervals from 1 to 14 dpi, 6 × 1 cm leaf sections were harvested from all treatments. Three of these were stained with BacLight green for bacteria and counterstained with propidium iodide. Stained leaves were imaged by confocal microscopy and the green percentage of each image used as a proxy for bacterial coverage (**A**). The remaining 3 leaf sections were homogenised in 10 mM MgCl_2_ and the homogenate spread onto KB-CFC agar to select for *Pseudomonas *sp. Colony counts were used to estimate the size of the *Pseudomonas* population (**B**). Values are means of two independent experiments and error bars show standard error. Letters above bars indicate significant (α = 0.05) differences between treatments (ANOVAs with Tukey’s simultaneous comparisons) and only apply within time points. No letters are shown where the ANOVA result was non-significant (**A**: 7, 10 dpi).

To discover whether the bacterial population on wheat leaves is affected by the presence of *Z. tritici*, leaves were inoculated with INA+ or INA− bacteria, frost treated, then either mock- or co-inoculated with *Z. tritici*. Epiphytic bacterial populations were assessed by (1) staining leaves with BacLight green and calculating percentage green-fluorescent area in surface micrographs (Fig. 3A) and (2) macerating leaf tissue and counting colonies recovered on *Pseudomonas* selective media (Fig. 3B). For both methods, controls without bacterial inoculation were used to provide a baseline for comparison, as leaves were non-sterile. Fluorescence measurements showed a significant increase in bacteria in inoculated *vs.* control samples at 24 h, but no effect of *Zymoseptoria*. By 4 dpi, both bacterial inoculum and *Zymoseptoria* had a significant effect, with larger increases in bacterial populations on co-infected leaves, particularly for INA+ bacteria. These differences disappeared by 7 dpi, with bacteria populations in all samples greatly reduced at 10 dpi. However, by 14 dpi, bacterial populations recovered, with both bacterial inoculum and *Zymoseptoria* once again significant due to particularly strong revival of INA+ populations on co-inoculated leaves (Fig. 3A). Colony counts confirmed the main results of the staining experiment. Larger bacterial populations were recovered from inoculated leaves, with larger bacterial populations on leaves co-inoculated with *Zymoseptoria* (detected at 7 dpi rather than 4 dpi). By 10 dpi, bacterial populations had fallen by at least an order of magnitude, but recovery in bacterial populations was seen at 14 dpi, with the most colonies recovered from leaves inoculated with INA+ bacteria and *Zymoseptoria* (Fig. 3B).

**Figure 4:**
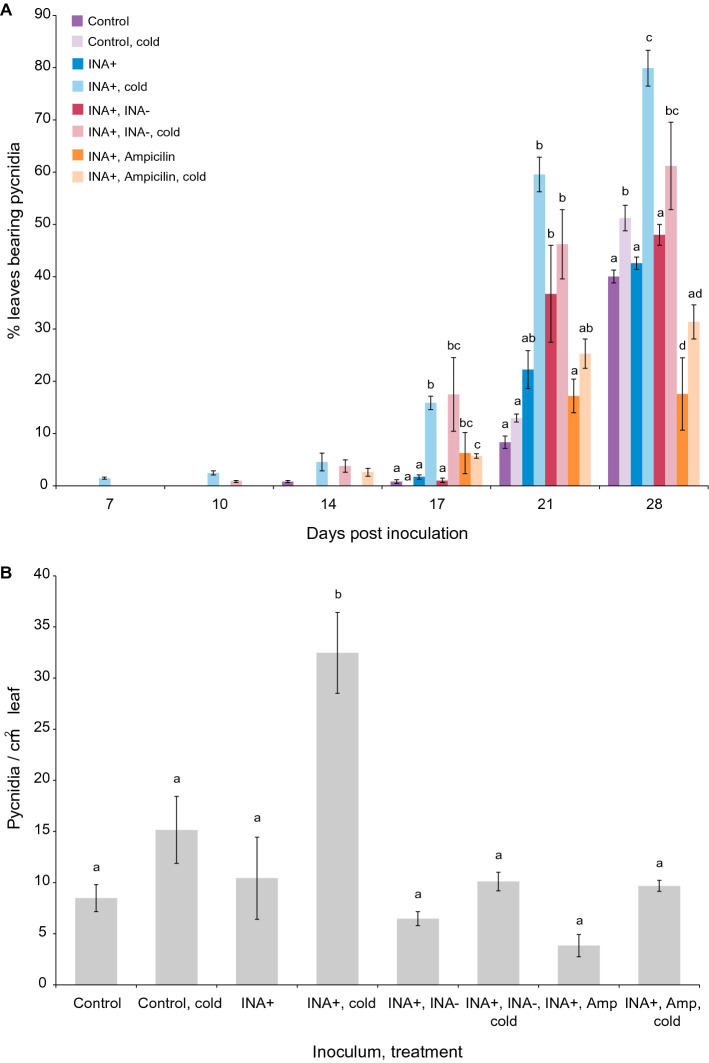
**Effect of controlling the population of INA+ bacteria on percentage of leaves inoculated with** ***Z. tritici*** **showing pycnidia at various time points after infection** (**A**) **and final pycnidial density** (**B**). Wheat leaves were inoculated with MgCl_2_ only (control, 24 pots), or with ice-nucleating bacteria (INA+) in MgCl_2_ (72 pots). Three to five leaves were inoculated in each pot. After 1 h drying, 24 INA+ inoculated pots were inoculated with non ice-nucleating (INA−) bacteria, and another 24 pots sprayed with 50 μg/mL ampicillin. After a further 24 h, 12 pots from each treatment were exposed to − 2 °C for 2 min. Pots were randomly assigned to treatments. All plants were then inoculated with *Z. tritici* blastospores at 10^5^ cfu/mL and infected plants maintained as normal. Leaves were inspected and numbers of leaves showing pycnidia recorded every 3–4 days from 7 to 21 dpi (**A**). At 28 dpi, leaves were harvested and pycnidia enumerated (**B**). Values are means of two independent experiments and error bars show standard error. Letters above bars indicate significant (α = 0.05) differences between treatments (ANOVAs with Tukey’s simultaneous comparisons) and only apply within time points in (**A**). No letters are shown where the ANOVA result was non-significant (**A**: 7, 10, 14 dpi).

To determine whether STB might be controlled by controlling epiphytic INA+ bacteria, wheat leaves inoculated with INA+ bacteria were sprayed with either ampicillin (antibiotic) or INA− bacteria (competitor) at 24 hpi. These leaves were frost-treated and inoculated with *Z. tritici* after a further 24 h. Symptom development occurred faster (from 7 dpi; Fig. 4A), and final coverage with pycnidia was higher (Fig. 4B), in INA+ inoculated, frost-treated plants, as previously. This acceleration of STB disease was not mitigated in plants treated with competitors. However, antibiotic treatment of INA+ inoculated leaves reduced STB incidence to control levels at 21 dpi. At 28 dpi, antibiotic-treated leaves showed a lower incidence of STB symptoms than control leaves.

## Discussion

This work demonstrates that frost damage can increase the speed and severity of STB*.* Such damage is likely to occur naturally under the conditions experienced by winter wheat in the UK^7^. It is reasonable to infer that the increased speed of symptom development is attributable to a decrease in the time required for randomly growing *Z. tritici*^8^ to penetrate the wounded leaf, due to the additional entry points provided by frost damage wounds. This would reduce the overall time required for the fungus to produce pycnidia. Similarly, the increase in pycnidiation on leaves exposed to INA+ bacteria and frost suggests that when more fungi penetrate the leaf early in infection, more extensive internal colonisation occurs. This relative underperformance of *Z. tritici* on unwounded leaves can again be explained by the previously described^8^ random fungal growth and entry. Early-penetrating hyphae, progressing through the wheat-*Zymoseptoria* interaction, hijack wheat’s anti-biotroph defences to promote necrotrophic infection^10,11^. This is likely to reduce colonisation by later-penetrating individuals that are still in the biotrophic phase. An increase in the synchronicity of penetration on wounded leaves might therefore explain the greater final pycnidial density seen. It is also possible to propose additional mechanisms for increased pycnidiation or faster disease development. Epiphytic fungal growth may be promoted by nutrients exuded from damaged leaves^17,19^. Frost damage is also likely to induce wound-signalling via jasmonate (JA) or ethylene (ET)^20^, which may benefit early, epiphytic or biotrophic growth of *Z. tritici*^21^ However, this would be detrimental to later, necrotrophic growth^10,21^. Since wounding occurred prior to *Z. tritici* inoculation, wound-induced JA-signalling may have promoted biotrophic infection. This implies that the characterisation of *Z. tritici* as a hemibiotroph is more accurate, at least for IPO323 (the isolate used in this study; see “Methods”), than the alternative ‘latent necrotroph’ description^22^. Further investigation of the impact of wound signalling upon STB development could help to clarify this key question of *Z. tritici* biology.

The impact upon STB disease of INA+ bacteria and of their control may open new avenues for crop protection against STB that do not rely on rapidly out-evolved fungicides^5^. Application of antibiotics to wheat fields is obviously unwise; however, other control methods, such as competitors, should be considered. Many such biocontrol applications do not persist on the leaf, making the timing of their application critical^23,24^. Possibilities include targeting the application of competitor bacteria to late frosts events where there is most leaf surface available to frost damage. This might reduce early establishment of *Z. tritici* on vulnerable crops in spring. Alternatively, bio- or other control methods might be used in combination with models that predict ascospore influx or ideal weather conditions for STB establishment, to avoid frost damage during high disease pressure. Such possibilities are of course dependent upon frost causing damage to the somewhat tougher leaves of older plants, since the experiments reported here have been carried out upon young wheat. However, it is known that UK wheat crops may be exposed to frosts as late as May, which do cause significant damage to the spikelets of at least the more frost-susceptible wheat varieties; whether the leaves are similarly affected was not reported^25^. It remains possible that these findings will only be field-relevant where frost-susceptible wheat is chosen, and more investigation on this point is needed. It would be of interest to compare historical data on the incidence of disease with that of frosts in April and May, especially where the wheat variety is known.

Data presented here indicate a possible mutualistic relationship between *Z. tritici* and *Pseudomonas syringae*, with an increase in bacterial populations in the presence of *Z. tritici*. Notably, this increase was larger and more consistent for INA+ *Pseudomonas*. There are many potential explanations for this relationship: bacterial or fungal induction of epiphytic nutrient exudation, sharing of secreted enzymes or siderophores, or plant defence suppression/induction, for example. Bacterial populations on all inoculated plants showed a decline after 4–7 dpi, leading to very low populations by 10 dpi. Colony counts indicate that both INA− and INA+ bacteria persisted better, until 7 dpi, when *Z. tritici* was also present. Until 7 dpi, most *Z. tritici* is epiphytic; it is therefore possible that the relationship between *Z. tritici* and *Pseudomonas syringae* requires physical contact or proximity, or reflects changes to their shared environment such as increased nutrient exudation. However, fluorescence data show this increased persistence of *Pseudomonas* only until 4 dpi, indicating that some of the colonies recovered at 7 dpi may have arisen from apoplastic bacteria. Increased bacterial success in the apoplast would indicate that epiphytic and/or biotrophic *Z. tritici* alters plant defences to benefit the biotrophic growth of *P. syringae.* Again, this supports the description of early invasive growth by *Z. tritici* as biotrophic and of *Z. tritici* as a hemibiotroph.

Both datasets show a recovery in INA+ bacterial populations at 14 dpi, exclusively in the presence of *Z. tritici*. This is not seen for INA− bacteria and indicates that the presence of *Z. tritici* enhances survivorship or promotes the regrowth of INA+ bacterial populations at or after 10 dpi. At 10–14 dpi, *Z. tritici* has entered the wheat leaf apoplast and is beginning to hijack plant anti-biotroph defences in order to promote its own switch to necrotrophy^7,10^. This is detrimental to co-infecting biotrophs, but is likely beneficial to necrotrophs and hemibiotrophs, as long as these are able to switch to necrotrophy concurrently with *Z. tritici*. Thus, the increase in INA+ colony counts might indicate that these bacteria have been more able to enter the leaf than INA− strains, allowing them to reach the necessary apoplastic quorum^26^ in time to switch to necrotrophy alongside the fungus. However, the increase in INA+ populations detected by fluorescence microscopy indicates that a portion of the increase was epiphytic. This is likely to be due to increased nutrient leakage from wounded leaves during the onset of necrotrophy, when nutrients are released from dying plant cells. Since ion leakage is heaviest from leaves treated with INA+ bacteria, it is reasonable to assume that epiphytic INA+ bacteria have increased access to this nutrient release.

It is important to note that INA+ bacteria used here were not isolated from wheat, so have not co-evolved with *Z. tritici*, although they may well have co-evolved with other plant-associated microbes. Sampling of bacterial and fungal populations from wheat leaves in the field is now necessary and will provide an exciting opportunity to understand plant pathology on a very broad scale: understanding the interactions between *Z. tritici* and INA+ bacterial epiphytes could help to clarify aspects of the pathogenesis and life cycle of *Z. tritici,* as well as providing a first step in better understanding the agro-ecology of the wheat leaf surface, and how this influences the evolution of INA+ bacteria, which are found worldwide in a huge variety of ecological settings^27^. In addition, the wheat-*Z. tritici-Pseudomonas* interaction is a promising model for studying the effect of the leaf microbiome on crop disease and for developing new, non-fungicide, control methodologies.

## Methods

### Plants

Wheat (variety Galaxie) was sown on John Innes No. 2 compost in 24-cell trays with 2–5 plants per cell, and maintained on a 16:8 h light:dark cycle at 18 °C (day) and 15 °C(night), with 80% RH in a MLR-352H-PE climate chamber (Sanyo). All plants were used for experiments at 2 weeks old.

### Bacteria and fungi

*Pseudomonas syringae* pv. syringae strains reported to exhibit ice nucleation activity (281, 3010) or not reported to exhibit ice nucleation activity (1902, 3012) were purchased from the National Collection of Plant Pathogenic Bacteria (FERA, UK). Strains selected were not originally isolated from wheat (281, 1902—isolated from *Syringa vulgaris*; 3010, 3012—isolated from *Malus sylvestris*) and are not known wheat pathogens. Ice-nucleation activity was confirmed by floating smooth foil squares on the surface of a water/methanol ice bath, cooled to − 10, − 8, − 6, − 4 or − 2 °C. 100 μL droplets of sterile, distilled water were pipetted onto the foil squares and ice crystals formed spontaneously, within 1 min, only at − 10 °C. Two microliters of overnight bacterial culture were added to water droplets and time taken for ice formation recorded. Strain 281 showed the strongest ice nucleation activity and 1902 showed no detectable effect on ice formation; 3010 and 3012 were intermediate (Supplementary Table S1). Bacteria were maintained in 50% glycerol at − 80 °C and were grown on LB agar at 28 °C for all applications. For all experiments involving *Z. tritici*, the model isolate IPO323^7^ was used.

### Bacterial pathogenicity tests

Bacteria were streaked onto LB agar and grown for 3 days before resuspension in 10 mM MgCl_2_ at 10^7^ cfu/mL. Bacterial suspensions were sprayed onto wheat plants using a hand held atomiser at a rate of approximately 0.5 mL per cell of 2–5 two week old plants, giving visible misting of both leaf surfaces, on all leaves, without runoff of inoculum. Six cells of plants were inoculated with each strain. Plants were then returned to growth chambers and monitored for symptom development for 28 days. Only strain 3010 induced clear symptoms within this timeframe, although some plants inoculated with strain 3012 also showed mild chlorosis of leaf tips after day 14 (Supplementary Table S2). In further experiments, only strains 281 (INA+) and 1902 (INA−) were used.

### Ion leakage measurements

10 cm lengths of 6–9 treated leaves were excised and placed in 10 mL ddH_2_O for 12 h. Conductivity of the ddH_2_O was measured using a conductivity meter and then leaves were boiled for 1 h and measurement repeated. ddH_2_O controls, without leaves, were treated in the same fashion. Ion leakage was reported as a percentage of total conductivity after boiling; control values were subtracted.

### Propidium iodide staining for cell death

1 cm leaf sections were immersed for 1 h, in the dark, in 0.05% (w/v) propidium iodide (PI), mounted in 0.1% (v/v) phosphate buffered saline (PBS, pH 7) and viewed using a Leica SP8 confocal microscope using argon laser emission at 500 nm with detection at 600–630 nm. Five leaf sections were viewed for each treatment and 3 fields of view visualised in each leaf section. Cell death was scored as number of cells showing internal (cytoplasmic or nuclear) red fluorescence / total number of cells in field of view. No cell death was recorded.

### Wheat inoculation

14 day-old wheat plants were inoculated with either INA+ or INA− bacteria suspended in 10 mM MgCl_2_ at 10^7^ cfu/mL by spraying with a handheld atomiser until leaves were visibly beaded with moisture on both surfaces, avoiding runoff. Controls were sprayed with 10 mM MgCl_2_ only.

For assays involving co-inoculation of plants with bacteria and *Z. tritici*, plants were allowed to dry for an hour before inoculation with *Z. tritici* blastospores. Blastospores were suspended in sterile distilled water at 10^5^ cfu/mL, a low inoculum density preventing saturation of infection^28^. Inoculated plants were returned to growth chambers and kept under plastic cloches for 72 h, then maintained as usual.

### Analysis of STB speed and severity

Plants were observed at 7, 10, 12, 14, 16, 18, 21, 24 and 28 days post inoculation (dpi) and the most severe symptom on each leaf recorded. At 28 dpi, all inoculated leaves were harvested, rehydrated for 1 h, then scanned at high resolution. Pycnidia were enumerated and leaf area measured in scanned images using ImageJ^28^.

### Antibiotic and competitor application

INA+ bacterial inoculation was carried out as before. Plants were allowed to dry for 1 h at room temperature, then spray inoculated with INA− bacteria suspended in 10 mM MgCl_2_ at 10^7^ cfu/mL, or sprayed with ampicillin solution (50 μg/mL). Following this second treatment, plants were returned to growth chambers for 24 h. Freezing treatment and subsequent *Z. tritici* inoculation was then carried out as above.

### Estimation of bacterial populations

Two methods were used to estimate bacterial populations on leaves. Firstly, 1 cm leaf samples were harvested from 3 randomly selected leaves in each treatment. These samples were mounted on glass slides in phosphate buffered saline, to which BacLight Green bacterial stain and propidium iodide counterstain were added at 0.05% (w/v) each. After 10 min, leaf samples were imaged at 20× magnification using a Leica SP8 confocal microscope. 5 μM z-stacks were collected at three randomly selected fields of view for each leaf sample and maximum projections created from these. Laser power, gain, and other parameters were held constant between fields of view and samples. The number of green pixels in each projection was then counted using ImageJ software and summed across the three fields of view. The percentage of pixels in the three fields of view which were green was then calculated and used as a proxy for percentage leaf area covered by bacteria. Secondly, 1 cm leaf samples were harvested from 3 more randomly selected leaves in each treatment and homogenised in 10 mM MgCl_2_. Homogenate was diluted 1/10, 1/100 and 1/1000 in MgCl_2_ and five 10 μL samples of each dilution spotted onto King’s B^29^ agar with *Pseudomonas* selective supplement CFC (Oxoid). Colonies were counted after 24 h incubation at 28 °C. Both procedures were carried out at 1, 4, 7, 10 and 14 dpi.

### Experimental design and statistical methods

Specific details of experimental design and analyses are presented in the figure legends alongside the relevant results. Some general principles were applied. Randomisation: where a set of pots of plants was divided between treatments, pots were numbered and randomly generated numbers used to select those assigned to each treatment. For selection of microscope fields of view within a leaf, the slide was placed so that the ‘bottom left’ part of the leaf was in view and a random distance (in mm, bounded by length and width of the leaf) moved in the x and y dimensions to select each field of view, returning to the 0,0 position before each selection.

Replication: technical replication was used in all experiments, with the number of such replicates given in each figure legend. Three complete repeat experiments were carried out in most cases, with figure legends stating where replication was different (min. 2 repeats) and all data presented represent the mean of such replicates, with error bars showing standard errors. Statistical analyses: data were analysed using ANOVA unless otherwise stated, with appropriate checks for homoscedasticity and other assumptions.

## Supplementary information

Supplementary Information
