# Supplementary material for: Presence of ice-nucleating Pseudomonas on wheat leaves promotes Septoria tritici blotch disease (Zymoseptoria tritici) via a mutually beneficial interaction
Source: Sci Rep. 2020 Oct 20;10:17738. doi: 10.1038/s41598-020-74615-7 (PMC7575590; doi:10.1038/s41598-020-74615-7)
Supplement: Supplementary file 1 — Supplementary Information [file 41598_2020_74615_MOESM1_ESM.pdf]

Presence of ice-nucleating *Pseudomonas* on wheat leaves promotes Septoria Tritici Blotch disease (*Zymoseptoria tritici*) *via* a mutually beneficial interaction.

Author: Fones, Helen N.[1]

1. Biosciences, University of Exeter, Stocker Road, Exeter, EX4 4QD
Correspondence to: h.n.eyles@exeter.ac.uk

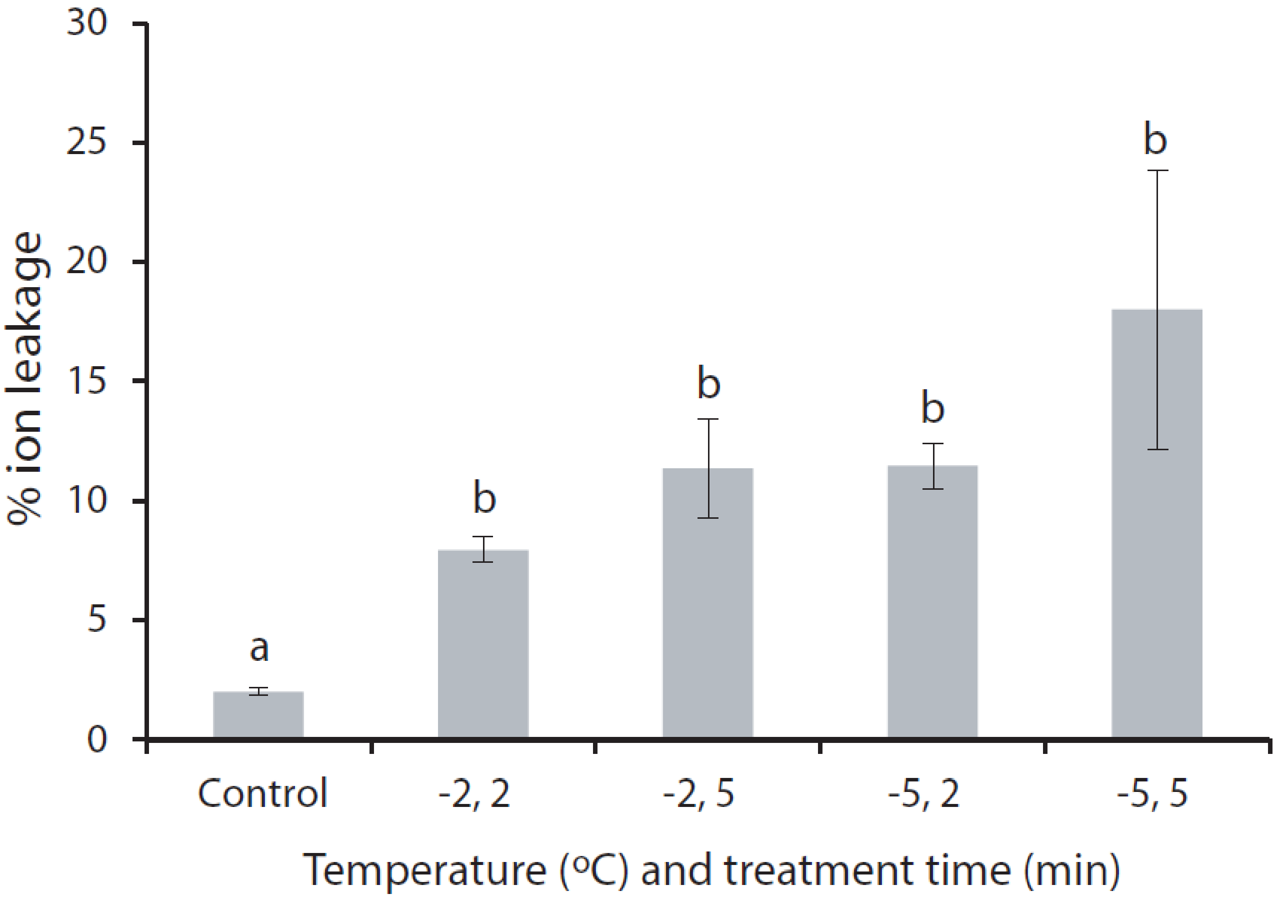

Figure S1: Frost treatment leads to increased ion leakage from wheat leaves. Leaves were subjected to 2 or 5 mins at -2 °C or -5 °C. Ion leakage from leaves increased significantly after freezing, compared to unfrozen control (ANOVA, $P = 0.046$, with Tukey's simultaneous comparisons; treatments that are significantly different at $\alpha = 0.05$ are indicated with different letters).

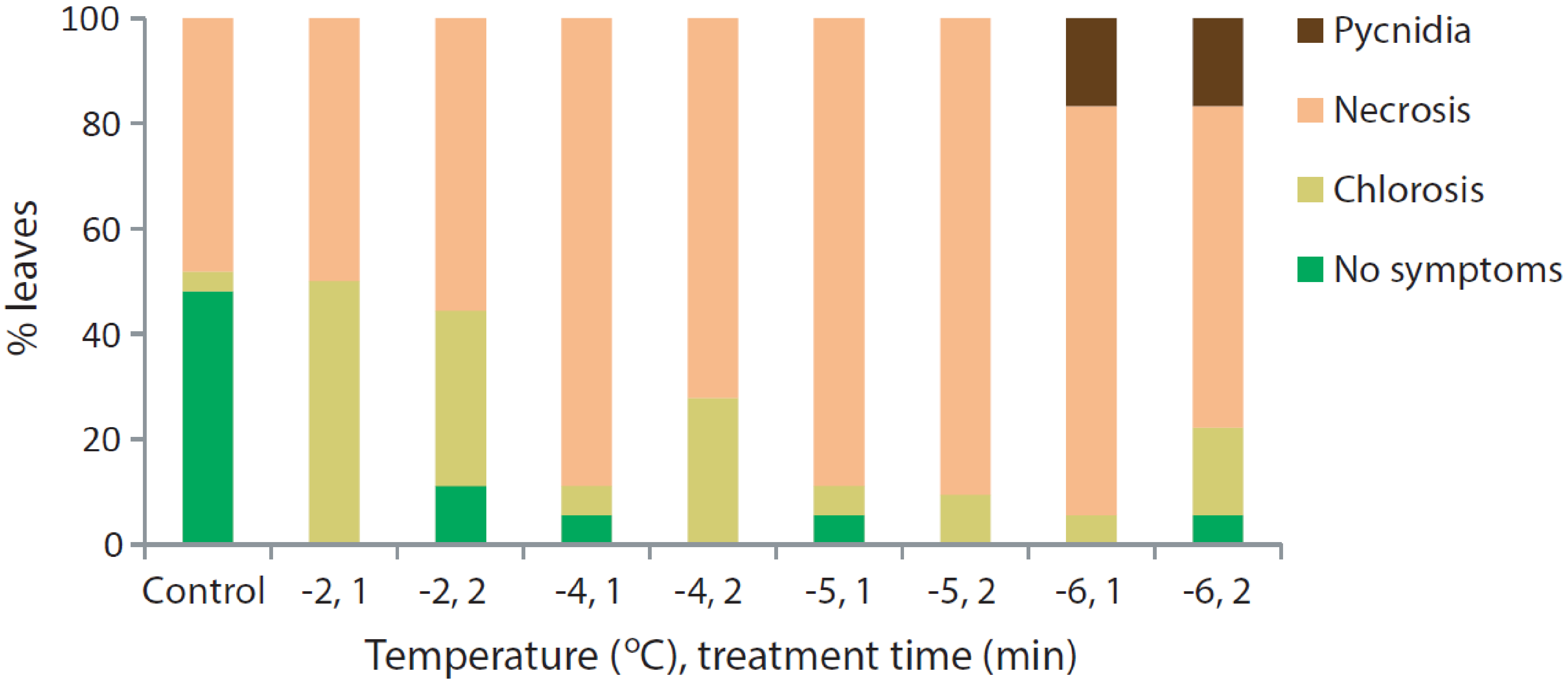

Figure S2: Frost treatment increases the speed and severity of STB disease on wheat. Leaves were subjected to 2 or 5 mins at the indicated temperatures. The most advanced disease symptom was on each leaf was scored and the percentage of inoculated leaves bearing that symptom is shown. Percentages are means of three independent experiments.

	Strain				
**Temp**	**None**	**281**	**3010**	**1902**	**3012**
**-10**	51.7	3.7	13.3	48.0	31.7
**-8**	> 60	5.3	14.0	47.3	32.0
**-6**	> 60	5.3	17.0	> 60	32.0
**-4**	> 60	8.7	45.0	> 60	> 60
**-2**	> 60	11.3	> 60	> 60	> 60

Table S1: Ice nucleation activity among tested strains. Seconds taken for 100 µL sterile distilled water (SDW), pipetted onto smooth foil floating on water/methanol at the stated temperatures, to freeze after 2 µL of bacterial culture (*Pseudomonas syringae* pv. syringae, labelled by NCPPB collection number) added (2 µL SDW for 'None' control). Strains reported as exhibiting ice nucleation activity in NCPPB catalogue shown in blue (281, 3010); strains not so reported shown in red (1902, 3012). Data are mean results from three independent droplet tests. 281 shows the strongest ice nucleating activity and only strain 1902 does not show shorter freezing times than control at -10 ^{o}C, the only temperature when 100 µL SDW froze within 1 min (*t*-test, P = 0.1).

	Leaf tip chlorosis					Whole leaf chlorosis					Necrosis (tip or lesions)				
	Strain					Strain					Strain				
dpi	None	281	1902	3010	3012	None	281	1902	3010	3012	None	281	1902	3010	3012
**2**	0	0	0	0	0	0	0	0	0	0	0	0	0	0	0
**4**	0	0	0	0	0	0	0	0	0	0	0	0	0	0	0
**7**	0	0	0	2.67	0	0	0	0	0.33	0	0	0	0	0.33	0
**10**	0	0	0	4.33	0	0	0	0	0.67	0	0	0	0	0.67	0
**14**	0	0	0	5.67	0	0	0	0	3	0	0	0	0	0.67	0
**21**	0	0	0	6	1.67	0	0	0	5.67	0	0	0	0	3	0
**28**	0	0	0	6	3	0	0	0	6	0	0	0	0	4.67	0

Table S2: bacterial pathogenicity on wheat var. Galaxie. Bacteria were spray inoculated onto 6 pots of wheat plants at 10^7 cfu/ml. Data represent number of pots with plants showing the given symptom at various time points up to 28 dpi and are averages of three independently inoculated sets of 6 pots. Cotyledons not assessed to avoid confusion between symptoms and senescence.
